# Reduced Cingulate Gyrus Volume Associated with Enhanced Cortisol Awakening Response in Young Healthy Adults Reporting Childhood Trauma

**DOI:** 10.1371/journal.pone.0069350

**Published:** 2013-07-24

**Authors:** Shaojia Lu, Weijia Gao, Zhaoguo Wei, Weiwei Wu, Mei Liao, Yuqiang Ding, Zhijun Zhang, Lingjiang Li

**Affiliations:** 1 Mental Health Institute, The Second Xiangya Hospital, Key Laboratory of Psychiatry and Mental Health of Hunan Province, Central South University, Changsha, Hunan, China; 2 Department of Psychiatry, Shenzhen Kangning Hospital, Shenzhen, Guangdong, China; 3 Key Laboratory of Arrhythmias, Ministry of Education, East Hospital, Tongji University School of Medicine, Shanghai, China; 4 The Department of Neuropsychiatry and Institute of Neuropsychiatric Research Affiliated ZhongDa Hospital of Southeast University, Nanking, Jiangsu, China; 5 Department of Psychiatry, Chinese University of Hong Kong, Hong Kong, China; Institution of Automation, CAS, China

## Abstract

**Background:**

Preclinical studies have demonstrated the relationship between stress-induced increased cortisol levels and atrophy of specific brain regions, however, this association has been less revealed in clinical samples. The aim of the present study was to investigate the changes and associations of the hypothalamic-pituitary-adrenal (HPA) axis activity and gray matter volumes in young healthy adults with self-reported childhood trauma exposures.

**Methods:**

Twenty four healthy adults with childhood trauma and 24 age- and gender-matched individuals without childhood trauma were recruited. Each participant collected salivary samples in the morning at four time points: immediately upon awakening, 30, 45, and 60 min after awakening for the assessment of cortisol awakening response (CAR). The 3D T1-weighted magnetic resonance imaging data were obtained on a Philips 3.0 Tesla scanner. Voxel-based morphometry analyses were conducted to compare the gray matter volume between two groups. Correlations of gray matter volume changes with severity of childhood trauma and CAR data were further analyzed.

**Results:**

Adults with self-reported childhood trauma showed an enhanced CAR and decreased gray matter volume in the right middle cingulate gyrus. Moreover, a significant association was observed between salivary cortisol secretions after awaking and the right middle cingulate gyrus volume reduction in subjects with childhood trauma.

**Conclusions:**

The present research outcomes suggest that childhood trauma is associated with hyperactivity of the HPA axis and decreased gray matter volume in the right middle cingulate gyrus, which may represent the vulnerability for developing psychosis after childhood trauma experiences. In addition, this study demonstrates that gray matter loss in the cingulate gyrus is related to increased cortisol levels.

## Introduction

Early life stress, including childhood trauma, which is very common in our society, has been established as a great risk factor in the subsequent development of multiple psychiatric disorders and unfavorable behavior patterns [1]. Previous studies have investigated the possible pathways from early life stress to psychosis, however, to date, little is known about the mechanisms underlying this association [2]. Recently, biological mechanisms such as dysfunction of the hypothalamic-pituitary-adrenal (HPA) axis [3] and altered volumes of specific brain regions [4] after exposures to early life stress have been reported which may help to elucidate the close relationship between early life stress and onset of psychosis.

Although not always consistent, findings for hyperactivity of the HPA axis as indicated by increased cortisol and adrenocorticotropin-releasing hormone (ACTH) responses to a psychological stress task [5] and to the dexamethasone/corticotropin-releasing factor (Dex/CRF) test [3], hypersecretion of salivary cortisol over the daytime hours [6], and enhanced cortisol awakening response (CAR) [7] among individuals with early life stress independent of psychosis diagnosis have been observed in an emerging body of investigations. With regard to anatomical magnetic resonance imaging studies, increasing evidence has shown a strong link between decreased hippocampus volume and early life stress [4], [8], [9]. Moreover, childhood emotional maltreatment was reported to be associated with profound reductions of medial prefrontal cortex (mPFC) volume [10] and in another study early deprivation was revealed to be correlated with reduced orbital-frontal cortical volume in postinstitutionalized children [11]. In line with these, the extent of gray matter loss in the cingulate cortex was suggested to be related to a history of early adverse events in a group of depression patients as well, although the sample size of the experimental group was relatively small [12].

It has been well known that glucocorticoids have neurotoxic effects in the central nervous system in some circumstances [13]. In addition, it is quite interesting that the altered brain regions reported in subjects with early life stress contain high concentrations of glucocorticoid receptors and act as well- documented roles in regulating HPA activity [14]. Hence, stress-related dysfunction of the HPA axis and atrophy of the regulator regions may precipitate a vicious circle which will result in greater exposure to glucocorticoids and more severe damage to these brain regions. This relationship has been revealed in preclinical studies [15], but it is less explored in clinical samples. Therefore, the aim of the present study was to investigate the changes and associations of the HPA activity and gray matter volumes in young healthy adults with self-reported childhood trauma exposures. To assess the HPA axis activity, the CAR, a reliable biological marker of reflecting the dynamic activity of the HPA axis [16], was administered to evaluate morning cortisol release to awaken. Based on previous findings, we hypothesized that childhood trauma could induce hyperactivity of the HPA axis and decreased gray matter volume in brain regions such as hippocampus, PFC, or cingulate gyrus; and there could be associations between increased cortisol concentrations and atrophy of those brain regions.

## Methods

### Participant

The study group comprised 48 subjects (male/female, 18/30), ages 18–33 years, including 24 subjects with childhood trauma experiences (CT group) and 24 age- and gender- matched subjects without childhood trauma exposures (non-CT group). For assignment to the CT group, individuals must have had experienced chronic moderate-severe trauma exposures (abuse or/and neglect) before the age of 16. All participants were recruited from a survey that we had carried out to investigate the occurrence of childhood trauma in local communities and universities. Subjects responded with no direct reference to childhood trauma as a key variable in the study. All subjects were thoroughly interviewed by two professional psychologists and were free from any current or lifetime history of psychiatric disorders according to Diagnostic and Statistical Manual of Mental Disorders, IV Edition (DSM- IV) criteria, as screened with the Structured Clinical Interview for DSM-IV interview (SCID). The general exclusions were as follows: (1) left handedness, (2) standard scores >50 on Zung's self-rating depression scale (SDS) [17] or >40 on Zung's self-rating anxiety scale (SAS) [18], (3) significant medical illness, (4) presence of major sensorimotor handicaps, (5) history of seizures, head trauma, or unconsciousness, (6) intake of any psychotropic medication or hormone, (7) alcohol or substance abuse, (8) women with pregnancy or in lactation or menstrual period, and (9) contraindications to MRI scan, including metallic implants, retractors or braces, and claustrophobia.

### Ethics Statement

This study was approved by the ethic committee of the Second Xiangya Hospital of Central South University. A complete description of the study was provided to every subject, after that written informed consent was obtained from each participant.

### Assessment of childhood trauma

The existence or absence of childhood trauma was determined by the childhood trauma questionnaire (CTQ) in all subjects. The CTQ is a 28-item retrospective self-report questionnaire designed to assess five types of negative childhood experiences by five sub-scales: emotional abuse, emotional neglect, sexual abuse, physical abuse, and physical neglect, respectively. Subjects who score higher than the threshold of a sub-scale are treated as existence of corresponding childhood trauma experience. The cutoffs of each sub-scale for moderate-severe exposure are: 1) emotional abuse ≥13, 2) emotional neglect ≥15, 3) sexual abuse ≥8, 4) physical abuse ≥10, and 5) physical neglect ≥10. This reliable questionnaire has great internal consistency and criterion-related validity in clinical and community samples; the good internal consistency coefficients for the five subscales are demonstrated in many studies [19], [20], [21], [22].

### Salivary cortisol

Salivary samples were collected on the magnetic resonance imaging scan day (weekdays) using Salivette® collection devices (Sarstedt, Nümbrecht, Germany) at altogether four time-points throughout the morning: immediately upon awakening, 30, 45, and 60 min following awakening. Samples were centrifuged at 3000 rpm (rounds per minute) for 10 min and recovered saliva samples were stored at −70°C until analysis. Participants are instructed not to brush their teeth and eat before completing sampling, or engage in heavy exercise during the collecting hour. Additionally, they were asked to refrain from smoking and from drinks except water [23]. Moreover, all subjects had to report the time they went to bed as well as the time they woke up [24].

The DRG® Salivary Cortisol ELISA Kit, SLV –2390 (Marburg, Germany) was used to measure salivary cortisol concentrations. The range of the assay is between 0.537–80 ng/ml. The intra- and inter-assay variability coefficients are 1.5–4.5% and 5.8–7.5%, respectively. As measures of the CAR, the area under the curve relative to ground (AUCg) and the area under the curve with respect to increase (AUCi) were calculated using the formulas described by Pruessner et al., (2003). The AUCg is a measure of the total cortisol secretion throughout the first hour following awakening, whereas the AUCi values reflect the dynamic of the CAR, which is more related to the sensitivity of the HPA system and focuses on the cortisol changes over time after awakening [25].

### MRI acquisition

Imaging data were acquired using a Philips 3.0-T scanner (Philips, Best, The Netherlands) in the Magnetic Resonance Center belonging to the Second Xiangya Hospital of Central South University. Subjects were asked to lie on the scanner and keep eyes closed. A standard birdcage head coil was used, and the restraining foam pads were placed on two sides of the head to minimize head motion while cotton plug was used with the purpose of diminishing the noise. For each subject, a high-resolution T1-weighted sequence using a three-dimensional magnetization prepared rapid acquisition gradient echo sequence was used. Images of the whole brain were acquired in an axial orientation with the following parameters: slice thickness  = 1 mm, gap  = 0 mm, repetition time  = 7.6 ms, echo time  = 3.7 ms, inversion time  = 795 ms, field of view  = 256*256 mm^2^, flip angle  = 8°, matrix size  = 256*256, resolution  = 1.0*1.0*1.0, slices  = 180, scan time  = 2′58′′.

### Voxel-based Morphometry (VBM) analysis

All T1-weighted high-resolution anatomical data were preprocessed by using the previous method [26], [27]. The analyses were performed using the Statistical Parametric Mapping 8 (SPM8) software (Wellcome Department of Imaging Neuroscience, University College London, UK; http://www.fil.ion.ucl.ac.uk/spm) in a Matlab environment. The VBM8 Toolbox (http://dbm.neuro.uni-jena.de/vbm.html) was used for preprocessing the structural images in SPM8 with default parameters. The data was bias-corrected, tissue classified, and normalized to Montreal Neurological Institute space using linear (12-parameter affine) and non-linear transformations within a unified model [28]. Then data analyses were performed on gray matter segment which was multiplied by the non-linear components derived from the normalization matrix in order to preserve actual gray matter value locally (modulated gray matter volume). Finally, the modulated gray matter volume was smoothed with a Gaussian kernel of 8 mm full width at half maximum.

### Statistical analysis

Data analyses were carried out using Statistical Package for the Social Sciences version 16.0 (SPSS Inc., Chicago, IL, USA). Independent two-sample *t* tests and Chi-square tests (*χ^2^*) were respectively used to tests for the continuous variables and categorical variables between the two groups. Salivary cortisol samples after awakening were analyzed using repeated measures ANOVA with time as the within-subjects factor and group as the between-subjects factor. The potential confounders, such as age, gender, time of awakening, hours of sleep, body mass index (BMI), and smoking, were included as covariates in the measures ANOVA to investigate possible effects on cortisol concentrations. Values are given as mean ± standard deviation. The level of two-tailed statistical significance was set at *p*<0.05 for all tests.

For gray matter volume, two-sample *t*-test on a voxel-by-voxel basis was performed to determine the difference between the two groups. The statistical threshold was set at *p*<0.05, corrected for multiple comparisons with false discovery rate (FDR) correction.

To evaluate any correlations between CTQ scores or CAR data and gray matter structural changes in individuals with childhood trauma, whole brain multiple regression analyses integrated in SPM basic models were performed at *p*<0.05 (FDR corrected). Moreover, Spearman rank correlation analysis was used to evaluate the relationship between CTQ scores and CAR data.

## Results

### Sample characteristics

As indicated in **Table 1**, the two groups of subjects did not differ with respect to age (*t* = −0.075, *p* = 0.940), gender (*χ^2^* = 0.000, *p* = 1.000), educational level (*t* = −1.407, *p* = 0.166), BMI (*t* = 1.839, *p* = 0.072), SAS score (*t* = 1.430, *p* = 0.160), SDS score (*t* = 1.014, *p* = 0.316), and smoking status (*Fish's exact test*  = 2.063, *p* = 0.609). As we would expect, the two experimental groups differed on levels of CTQ and its sub-scales except sexual abuse (*t* = 3.234∼11.38, *p*<0.01). The most common aspect of childhood trauma experience in the present sample was emotional neglect (17, 70.8%); a proportion of 62.5% (15) of traumatic subjects experienced at least two forms of childhood trauma exposures.

**Table 1:** Demographic and clinical characteristics of all subjects (n = 48).

	CT group, n = 24			non-CT group, n = 24		
	Mean	SD	Range	Mean	SD	Range
Age (Years)	21.5	3.98	18–33	21.5	3.69	18–33
Gender (Male/Female)	9/15			9/15		
Educational level (Years)	14.0	1.30	12–17	14.7	1.92	12–18
BMI (kg/m^2^)	21.6	2.05	17.7–25.0	20.6	1.62	18.3–23.3
SDS score	36.2	6.06	25–46	34.5	5.30	27–48
SAS score	34.0	4.51	26–40	32.0	4.78	25–40
Smoking, n (%)						
0	21(87.5)			22(91.7)		
≤ 10	1(4.17)			2(8.3)		
11–20	2(8.33)			0(0)		
CTQ score						
Emotional abuse**	9.21	2.36	6–15	6.21	1.22	5–9
Physical abuse**	7.83	2.93	5–14	5.71	1.33	5–9
Sexual abuse	5.46	0.83	5–7	5.38	0.58	5–6
Emotional neglect**	15.2	3.28	7–20	7.38	2.65	5–13
Physical neglect**	10.2	2.72	5–17	5.63	0.93	5–8
Total**	47.9	6.08	39–58	30.2	4.63	25–40
CT exposures, n (%)						
Emotional abuse	2(8.33)					
Physical abuse	8(33.3)					
Sexual abuse	0(0)					
Emotional neglect	17(70.8)					
Physical neglect	14(58.3)					
Multiply Exposures	15(62.5)					
Single Exposure	9(37.5)					

BMI, body mass index; CT, childhood trauma; CTQ, childhood trauma questionnaire; SAS, self-rating anxiety scale; SD, standard deviation; SDS, self-rating depression scale.

** *p*<0.01.

### Cortisol awakening response

**Figure 1:**
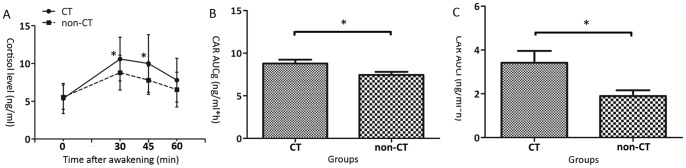
Changes in cortisol awakening response (CAR) across the experimental groups. (**A**) Independent two sample *t* tests revealed that significant differences of salivary cortisol levels were found between two groups at 30 (*t* = 2.389, *p* = 0.021) and 45 min (*t* = 2.565, *p* = 0.014) after awakening. Significant increases of cortisol levels were observed in subjects with childhood trauma experiences at those two time points. (**B**) The CAR area-under-the-curve to ground (AUCg) was significantly differed between two groups (*t* = 2.335, *p* = 0.024). Consistent with cortisol levels at 30 and 45 min, subjects who had self-reported childhood trauma showed higher levels of CAR AUCg. (**C**) With respect to the CAR area-under-the-curve increase (AUCi), significant difference was found as well (*t* = 2.532, *p* = 0.016). *Comparison with non-CT group, *p* <0.05. CT, childhood trauma.

There was no significant difference between two groups for time of awakening (CT group 6:46±19 min *vs* non-CT group 6:40±22 min, *p* < 0.05) or hours of sleep (CT group 7.04±0.69 h *vs* non-CT group 6.78±0.47 h, *p* < 0.05). A repeated-measures ANOVA of the morning salivary cortisol concentrations revealed a significant main effect of group (*F* = 5.111, *p* = 0.029), a main effect of time (*F* = 47.60, *p* = 0.000), and a group * time effect (*F* = 3.667, *p* = 0.014). There were no main or interactive effects of age, gender, BMI, smoking, time of awakening or hours of sleep on cortisol levels when introduced as covariates in the ANOVA. Independent two-sample *t* tests of the individual time points showed that salivary cortisol concentrations were significantly higher in traumatic subjects at 30 and 45 min following awakening (see **figure 1A**). Meanwhile, a statistically significant increase was observed for AUCg of salivary cortisol secretion in subjects who had self-reported childhood trauma experiences (see **figure 1B**). With respect to AUCi, the CT group also showed a higher level than the non-CT group with a statistically significant difference (see **figure 1C**).

### Voxel-based analysis of morphometry

**Figure 2:**
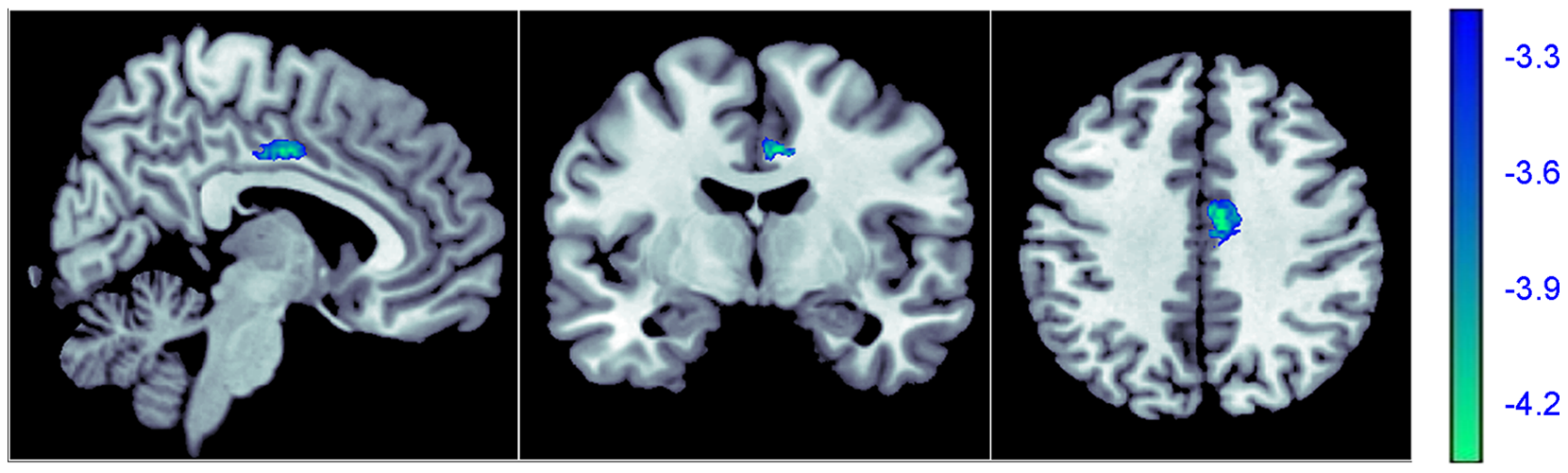
Region of different gray matter volume between individuals with and without childhood trauma. Decreased gray matter volume was detected in the right middle cingulate gyrus (Brodman area 24, *x* = 6, *y* = −6, *z* = 39, *cluster size*  = 303, *z score*  = 4.25, *p_uncorrected_*<0.001, *p_FDR corrected_*  = 0.047,) in subjects with childhood trauma. The right middle cingulate gyrus is shown in blue.

As compared with subjects without childhood trauma, individuals with adverse experiences in childhood showed significant volume reduction in the right middle cingulate gyrus (see **Figure** **2**). However, no region with significantly increased volume in traumatic subjects was observed.

### Correlations

In subjects with childhood trauma, the whole brain linear regression analysis conducted with SPM8 yielded a strong negative association of the right middle cingulate gyrus volume with CAR AUCg (see **Table 2**), however, there were no brain areas revealing significant correlations with CTQ scores at the defined threshold. Finally, a positive association was observed between CTQ total score and CAR AUCg (*r_s_* = 0.674, *p* = 0.000) in individuals with childhood trauma experiences.

**Table 2:** Region of decreased gray matter volume in subjects with childhood trauma correlated to CAR AUCg.

Anatomical region	BA	Side	Cluster size	x	y	z	z-score	*p*_uncorrected_	*p*_FDR corrected_
Middle cingulate gyrus	24	R	419	5	−6	43	4.24	<0.001	0.047

AUCg, area-under-the-curve to ground; BA, Brodman area; CAR, cortisol awakening response; FDR, false discovery rate.

## Discussion

The present study investigated the HPA activity as measured by CAR and brain structural (gray matter volume) changes in young healthy adults with and without childhood trauma experiences. The current results revealed a significantly enhanced CAR and decreased gray matter volume in the right middle cingulate gyrus (BA 24) and furthermore, a significant association between morning salivary cortisol levels after awaking and the right middle cingulate gyrus volume in subjects with childhood trauma. These outcomes together with the previous preclinical findings suggested that stress-related brain structural volume reductions might be the consequences of prolonged exposure to increased levels of glucocorticoids resulting from chronic early life stress [13].

The enhanced CAR in subjects reporting childhood trauma experiences was a main finding of the present study which indicated that childhood trauma seemed to be a good predictor of hyperactivity of the HPA axis even in the absence of current psychosis diagnosis. This indication was supported by prior studies evaluating the effect of childhood parent loss on adult HPA axis function [29] and investigating the relationship between childhood abuse and HPA axis function using a DEX/CRH test in female borderline personality disorder patients [30], both demonstrating the association between early life stress and hyperactivity of the HPA axis. Our neuroendocrine findings were also generally in accordance with a previous report in young healthy adults with low parental care experiences exhibiting an increased CAR and increased afternoon/evening cortisol outputs [7]. However, hyperactivity of the HPA axis in subjects with early life stress was not always replicated, both diminished cortisol responses in healthy adults reporting significant childhood maltreatment [31] and decreased CAR in healthy college students after early loss experiences [32] were observed in relevant studies. Several potential explanations have been posited to account for this inconsistency, such as sex differences, differences in the timing and severity of childhood trauma, genetic factors, and different concomitant and subsequent psychosocial conditions in later life [32], [33].

In the present study, the right middle cingulate gyrus gray matter volume reduction was detected in subjects with childhood trauma. The cingulate gyrus, a part of the limbic system, plays a critical role in two main neuroanatomic circuits that are believed to be involved in mood regulation [34]. The extensive connections of the cingulate gyrus with the frontal lobe, the temporal lobe and the striatal structures enable it to be an important area for the integration of emotions [35]. Our data was, at least in part, comparable with findings of reduced gray matter volumes in the cingulate gyrus in both healthy adolescents [36] and adults [4] with high CTQ scores. Interestingly, smaller gray matter volume in the cingulate gyrus was also present in patients with current or remitted depression [37], [38] and post traumatic stress disorder [39] in previous anatomical MRI studies which had focused on this region. In this regard, reduced cingulate gyrus volume before illness onset which contributed a limbic scar induced by long-term childhood trauma experiences in young healthy adults might represent the psychobiological vulnerability for developing psychosis.

It has been demonstrated that hypercortisolism induced by adrenalectomy with high-dose dexamethasone supplementation is associated with a significant reduction in the volume of the cingulate gyrus in rats [40]. In this context, the observed relationship between reduced cingulate gyrus and increased salivary cortisol secretion after awaking in our study extended that preclinical finding. As reported previously, the cingulate gyrus contains high densities of glucocorticoid receptors in most layers [14] which may make it one of the most vulnerable parts to the neurotoxicity of increased glucocorticoids. In addition, the cingulate gyrus also plays a crucial role in the regulation of the HPA axis [41], and thus glucocorticoids induced damage to this area may diminish its ability to exert negative feedback control resulting in more glucocorticoid secretions. This cycle may help to explain the origin of the present association in subjects with childhood trauma, however, our study was cross-sectional designed which precluded causal inferences. Further research will be required to clearly clarify this causal relationship.

Unexpected from our initial expectations, this study did not found any group differences of gray matter volume in hippocampus and PFC that have predominantly been linked to childhood trauma experiences. This may result from heterogeneity of research samples or types of childhood trauma experiences. Previous studies that have detected decreased gray matter volumes in hippocampus and PFC associated with childhood trauma experiences have measured this relationship in patients with depression [9] or in a mixed study sample [10], including healthy adults, patients with depression and/or anxiety disorder. Therefore, volume reductions in hippocampus and PFC may probably sever as results of co-appearance of childhood trauma and psychosis but not childhood trauma alone. In addition, in our sample, the subjects are young (average age = 21.5) which may restrain the time-dependent glucocorticoids induced atrophy of key brain regions. Finally, most studies reporting neuroanatomical correlates of childhood trauma with decreased hippocampus and PFC volumes in adults have focused mainly on the impact of sexual and/or physical abuse [42], [43], [44], but the most frequent aspects of childhood trauma experiences in our samples are physical and emotional neglect, which may also contribute to the differences mentioned above.

## Limitations

Several limitations must be taken into account when interpreting our findings. First, although it is suitable for CAR and VBM analyses, the sample size in each group is relatively small which restricts us to compare the different types of childhood trauma on HPA axis activity and brain structural changes. Second, childhood trauma experiences are evaluated by a retrospective self-reported questionnaire; hence, the results could be influenced by information biases. Finally, we cannot control for the subjects' compliance with the given sampling time schedule since a prior study has indicated that the participants' compliance with instructions is generally low [45], which may influence the CAR outcomes. However, our CAR results are characterized by a sharp increase in cortisol levels following awaken, peaking at 30 min later which well stands in line with other findings [46].

In conclusion, the present research outcomes suggest that childhood trauma is associated with hyperactivity of the HPA axis and decreased gray matter volume in the right middle cingulate gyrus, which may represent the vulnerability for developing psychosis after childhood trauma experiences. In addition, this study demonstrates that gray matter loss in the cingulate gyrus is related to increased cortisol levels.
